# Telomere-to-telomere characterization of rDNA chromosome in the myxomycete *Didymium iridis*

**DOI:** 10.1186/s12860-026-00587-7

**Published:** 2026-04-06

**Authors:** Arif Khan, Tor Erik Jørgensen, Lars Martin Jakt, Bård Ove Karlsen, Annica Hedberg, Steinar Daae Johansen

**Affiliations:** 1 Genomics Division, Faculty of Biosciences and Aquaculture, Nord University, Bodø, Norway; 2 Department of Laboratory Medicine, Nordland Hospital Trust, Bodø, Norway; 3 Department of Medical Biology, Faculty of Health Sciences, UiT the Arctic University of Norway, Tromsø, Norway

**Keywords:** Catalytic RNA, Direct repeat, Group I intron, Homing endonuclease mRNA, Myxomycete, Palindromic repeat, Spliceosomal intron

## Abstract

**Background:**

The ribosomal DNA (rDNA) of the myxomycete *Didymium iridis* is located on a linear, multi-copy, non-Mendelian chromosome. Efforts to determine the complete sequence by short-read sequencing technologies have been prevented by the presence of highly repetitive regions. Here we use high coverage (~10,000 x) long-read Oxford Nanopore Technology to determine the rDNA chromosome sequence in haploid amoebae from telomere-to-telomere.

**Results:**

The 20 kb rDNA chromosome, which is present at ~ 132 copies per haploid genome, is capped by regular TTAGGG telomeric repeats at both ends and carries an 11.3 kb pre-rRNA transcription unit coding for the small and large subunit rRNAs. The rRNA genes are further interrupted by autocatalytic group I introns, one of which encodes a homing endonuclease and two catalytic RNA domains with different functions in RNA processing. RNA mapping analyses from amoeba, microcyst, flagellate, and plasmodium stages, based on Illumina short-read sequencing, support the presence of a mature intron homing endonuclease mRNA both in haploid and diploid life stages in *D. iridis*. The non-transcribed sequence region upstream of the transcription unit contains several direct repeat arrays, including a highly complex upstream promoter region likely to be involved in pre-rRNA transcription regulation. Adjacent to the upstream telomere, a 4.2 kb palindromic region with potential for cruciform structure formation is found. Here, two putative replication origin candidates are located.

**Conclusions:**

High coverage Oxford Nanopore Technology sequencing results in excellent resolution of complex sequence repeat feature in the *D. iridis* rDNA chromosome. The rRNA genes are interrupted by complex group I introns and RNA sequencing supports intron autocatalytic processing in haploid and diploid life stages. This study provides new insights into structural arrangements of nuclear rDNA in eukaryotic microorganisms.

**Supplementary information:**

The online version contains supplementary material available at 10.1186/s12860-026-00587-7.

## Background

In multicellular eukaryotes, ribosomal DNA (rDNA) is organized as long tandem repeat arrays at one or more Mendelian chromosomes and contributes to the nucleolar structure organization [1]. These rDNA arrays serve as templates for the large amount of ribosomal RNA (rRNA) molecules needed in each cell cycle and are usually evolutionary unstable due to extensive homologous recombination [2, 3]. In unicellular eukaryotes, however, non-Mendelian chromosomal rDNA is widespread [4]. Examples include *Naegleria* amoebo-flagellates [5, 6], *Tetrahymena* ciliates [7, 8], *Dictyostelium* cellular slime molds [9, 10], and *Physarum* plasmodial slime molds (reviewed in [11]). While the *Naegleria* rDNA has a circular plasmid-like organization of approximately 15 kb, *Tetrahymena*, *Dictyostelium* and *Physarum* possess linear rDNA chromosomes of approximately 21 kb, 88 kb, and 60 kb in size, respectively. These chromosomes contain two rRNA transcription units forming a palindrome around a central region.

The rDNA of the plasmodial slime mold (myxomycete) *Physarum polycephalum* has been partially sequenced and investigated in some detail. The 60 kb rDNA chromosome is a giant palindrome that carries two rRNA transcription units and is present at approximately 150 copies per haploid nucleus. It is inherited in a non-Mendelian fashion and capped at both ends by regular TTAGGG telomeric arrays [12–15]. The 13.3 kb primary rRNA transcript is further processed into the small subunit (SSU; 19S) rRNA [16] and the large subunit (LSU; 5.8S + 26S) rRNAs [17]. The LSU rRNA gene is interrupted by two mandatory group I introns present in all isolates and strains investigated so far [18], and an optional mobile intron only found in the Carolina-isolate and derived strains [19, 20]. The central rDNA spacer, which separates the two diverging transcription units, is approximately 23 kb in size and loaded with complex patterns of inverted and direct repeats [21, 22]. The spacer architecture is still not fully understood, probably due to technical difficulties in generating contiguous sequences. However, it is known to carry four replication origins, two in each palindrome half, which are activated independently during the S-phase of the cell cycle [12, 21, 23].

*Didymium iridis* belongs to the same Physarales myxomycete order as *P. polycephalum* but classified in a different taxonomic family [24]. Like *P. polycephalum*, the *D. iridis* rDNA is located on a small, non-Mendelian, and multicopy chromosome [25]. Partial DNA sequencing analyses have shown that each *D. iridis* rDNA chromosome harbors a single rRNA transcription unit encoding the SSU and LSU rRNAs [26, 27]. Interestingly, both the SSU and LSU rRNA genes are interrupted by group I introns, and two mandatory LSU rRNA introns, like those in *P. polycephalum*, have been noted [18]. In contrast, the SSU rRNA gene harbors three optional introns. One intron (named S1389) is present in two of eight investigated isolates [28]. The two additional optional introns, named S956-1 and S956-2, are mobile genetic elements that self-splice in vitro as naked RNA [29, 30]. Both S956-1 and S956-2 contain homing endonuclease genes (HEGs), but in opposite orientations, and code for active DNA homing endonuclease (HE) proteins [30, 31]. The intron S956-1 (from the Pan 2 isolate) is reported to be mobile in genetic crosses between intron-containing and intron-lacking haploid amoebae strains [27]. Expression of protein-coding genes in a eukaryotic rDNA context is challenging and unusual [32]. The HE mRNA, however, undergoes 5’ capping, 3’ polyadenylation, spliceosomal intron removal, and is finally translocated to the cytoplasm for translation [33, 34]. The 5’ cap is comprised of a unique 3-nt lariat and is different in structure from the typical m7G cap found in most eukaryote mRNAs and is generated by a specific lariat-capping ribozyme located within the S956-1 intron [34, 35].

As part of an ongoing whole-genome sequencing project of the *D. iridis* Pan 2 isolate, we report the complete telomere-to-telomere sequence of the non-Mendelian rDNA chromosome. The rDNA includes a highly complex pattern of direct and inverted repeats, as well as an rRNA transcription unit carrying group I intron insertions. RNA isolated from haploid and diploid life cycle stages was mapped onto the rDNA chromosome, and an abundant polyadenylated mRNA was identified that corresponds to a group I intron homing endonuclease gene.

## Methods

### *D. Iridis* strains, cell culturing and nucleic acid extractions

**Fig. 1:**
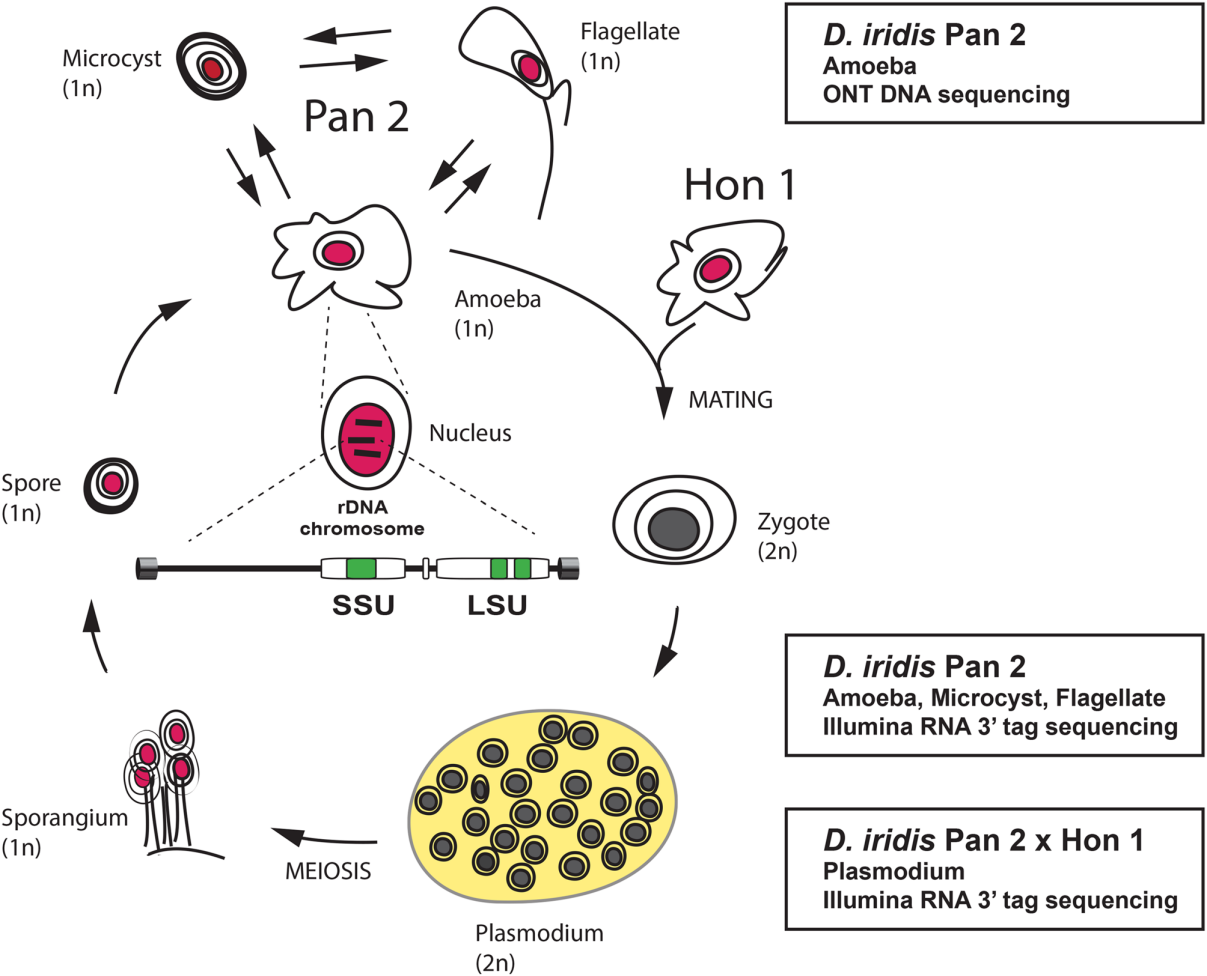
Life cycle of *D. iridis* and DNA/RNA sequencing strategy. Haploid amoebae (Pan 2 isolate) were differentiated into flagellates or microcysts. Pan 2 and Hon 1 amoebae carry different mating-type alleles and can be fused into a diploid zygote, which further develops into a multi-nuclei plasmodium cell (yellow). After extended starvation, a mature plasmodium can undergo meiosis and differentiate into a sporangium carrying haploid spores. Finally, a mature spore can germinate into a proliferating haploid amoeba. Haploid and diploid nuclei are indicated in red and dark grey, respectively. A schematic drawing of the rDNA chromosome is shown in the center. SSU, small subunit rRNA gene; LSU, large subunit rRNA gene. rDNA chromosome and transcriptome sequencing strategies, based on Oxford Nanopore technology (ONT) and Illumina 3’ tag sequence technologies, are indicated (right)

The myxomycete *D. iridis* isolates Pan 2 and Hon 1 [36] were used in this work, and strains Pan 2–16, Pan 2–44, and Hon 1–7 were originally provided by Dr. M. Silliker, Dr. G. Shipley, and Dr. J. Clark, respectively. *D. iridis* Pan 2–16, Pan 2–44, and Hon 1–7 are available from The American Type Culture Collection (http://www.atcc.org) at ATCC #66542, ATCC #66541, and ATCC #24464, respectively. The *D. iridis* life cycle and sequencing approach is summarized in Fig. 1. Strains were maintained and cultivated as previously reported [27, 37]. In short, *D. iridis* amoebae were grown at 26 °C in liquid DS/2 medium containing *E. coli* KB as food source. Haploid Pan 2–44 amoebae were differentiated into dormant microcysts when the bacterial food source became depleted. Excystment in free-water conditions results in non-dividing bi-flagellate swarm cells. Diploid plasmodium was generated by mating of Pan 2–44 and Hon 1–7 haploid amoebae. High-molecular total DNA was isolated from Pan 2–16 haploid amoeba using sodium perchlorate-treated cell lysates [38]. Total RNA was isolated from haploid amoeba, microcysts, and flagellates, and diploid plasmodia using the Trizol Reagent (ThermoFisher Scientific) as previously described for *D. iridis* cells [37, 39].

### DNA and RNA sequencing

The rDNA chromosome was sequenced along with the whole *D. iridis* Pan 2 genome (haploid amoebae) by Oxford Nanopore Technology (ONT). Library preparations were based on the SQK-LSK110 (ONT, Oxford, UK). Total DNA sequencing was carried out on three MinION Mk1C sequencer R9.4.1 flow cells (FLO-MIN106) according to the instructions given by the manufacturer (ONT, Oxford, UK). Raw sequence data was basecalled using Guppy version 6.2.1 with the super-accurate configuration, yielding a total of around 22 Gbp of sequence data. RNA library preparation and sequencing was provided as a commercial service by Eurofins MWG Operon (Ebersberg, Germany) and based on sequencing using the Illumina HiSeq™2000 platform. Here, 3’-tag libraries with preferences for polyadenylated RNA were made separately from the amoebae, microcyst, flagellate and plasmodium stages and subsequently sequenced.

### rDNA assembly

ONT sequences were assembled to a draft genome based on Flye (version 2.9.1-b1780) [40] using the nano-hq option (–nano-hq) and two polishing iterations (−i 2). The ONT sequences were mapped to the assembly using minimap2 (version 2.17-r974-dirty) piped to samtools to create sorted and indexed BAM (Binary Alignment Map) files. These and other BAM files were analyzed using the R plugin read_bam (https://github.com/lmjakt/Read_bam). We identified a contig containing the rDNA locus (contig_236) by mapping a partial assembly of the rDNA chromosome to the draft genome assembly using minimap2. We created an initial draft telomere-to-telomere assembly of the rDNA chromosome by manually combining sequences from contig_236 and the prior assembly. This assembly was polished by multiple sequence alignments of raw ONT reads (using the R msa package) that mapped to it, and by available Sanger sequences. The median read depth of chromosomal length contigs and the rDNA part of contig_236 was 87 and 11,588 respectively. This suggests a copy number of ~ 132 rDNA chromosomes per haploid genome. Direct and inverted repeats were identified using dot plots made using the string_plt R plugin (https://github.com/lmjakt/string_plotR) and these were used to guide a manual annotation of the sequence.

### RNA data analyses

Quality-filtering and contig assembly of Illumina reads were performed as a service from Eurofins MWG Operon (Ebersberg, Germany). The Illumina sequencing yielded in total 86.3 million quality-filtered reads, which represented 18.4, 27.9, 20.1, and 19.8 million reads from amoeba, microcysts, flagellates, and plasmodia, respectively. The Fastqc bioinformatics tool (version 0.12.0) [41] was used to check the quality of sequenced reads. Adapters and low-quality reads, including reads shorter than 40 bp, were removed using Trimmomatic (version 0.39) [42] keeping the Phred quality score (Q) ≥30. *De novo* assemblies from each life stage were generated by assembling all high-quality reads using the Trinity assembler (version 2.8.6) [43]. RNA reads from all stages were mapped onto the assembled rDNA chromosome using STAR (version 2.7.11b) [44]. Secondary folding patterns of ribozymes were based on reported structure assessments and predictions [35, 37, 45]. Structure presentations in figures were generated in Adobe Illustrator 2020 (http://www.adobe.com; last accessed October 2025).

## Results

### The *D. iridis* rDNA chromosome harbors complex patterns of inverted and direct repeats

**Fig. 2:**
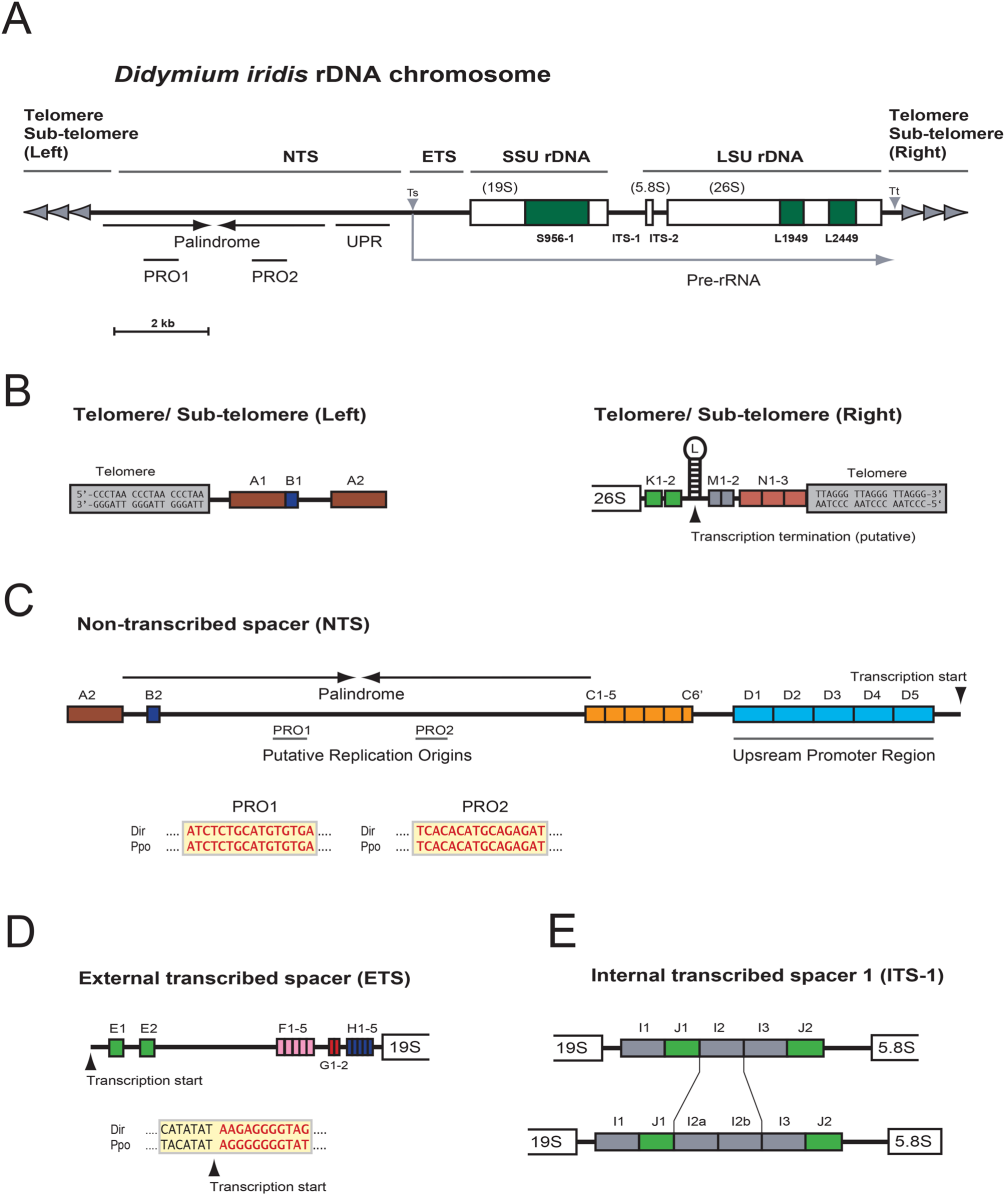
Schematic organization of the *D. iridis* rDNA chromosome in Pan 2 isolate. (**A**) the rDNA chromosome (20.3 kb) has a linear structure flanked by TTAGGG telomeres at the ends. The transcription unit (11.3 kb) contains the SSU and LSU rRNA genes. The precursor rRNA (pre-rRNA) is processed into a 19S rRNA (after removal of the mobile group I intron S956-1), a 5.8S rRNA, and a spliced 26S rRNA (removal of the group I introns L1949 and L2449). ETS, external transcribed spacer; ITS-1 and ITS-2, internal transcribed spacers 1 and 2; Ts and tt, transcription start site and termination site, respectively. NTS, non-transcribed spacer. The 4.2 kb palindromic structure contains putative replication origins (PRO1 and PRO2). UPR, upstream promoter region. (**B**) expanded chromosomal terminal regions. Repeat motifs A, B, K, L, M and N are indicated. (**C**) expanded structure of NTS. Repeat motifs A, B, C and D are indicated. Sequence comparison of the putative replication origins in *D. iridis* (Dir) rDNA to the known replication origins in *P. polycephalum* (ppo) rDNA. (**D**) ETS contains the repeat motifs E, F, G and H. Sequence of the putative transcription start in Dir rDNA is compared to the known site in ppo rDNA. (**E**) strain specific polymorphism in ITS-1. Repeat motifs I and J are indicated. While isolates Pan 2, Pan 3, and CR 8 contain three I-motif copies, Hon 1 and Gua1 have four

**Fig. 3:**
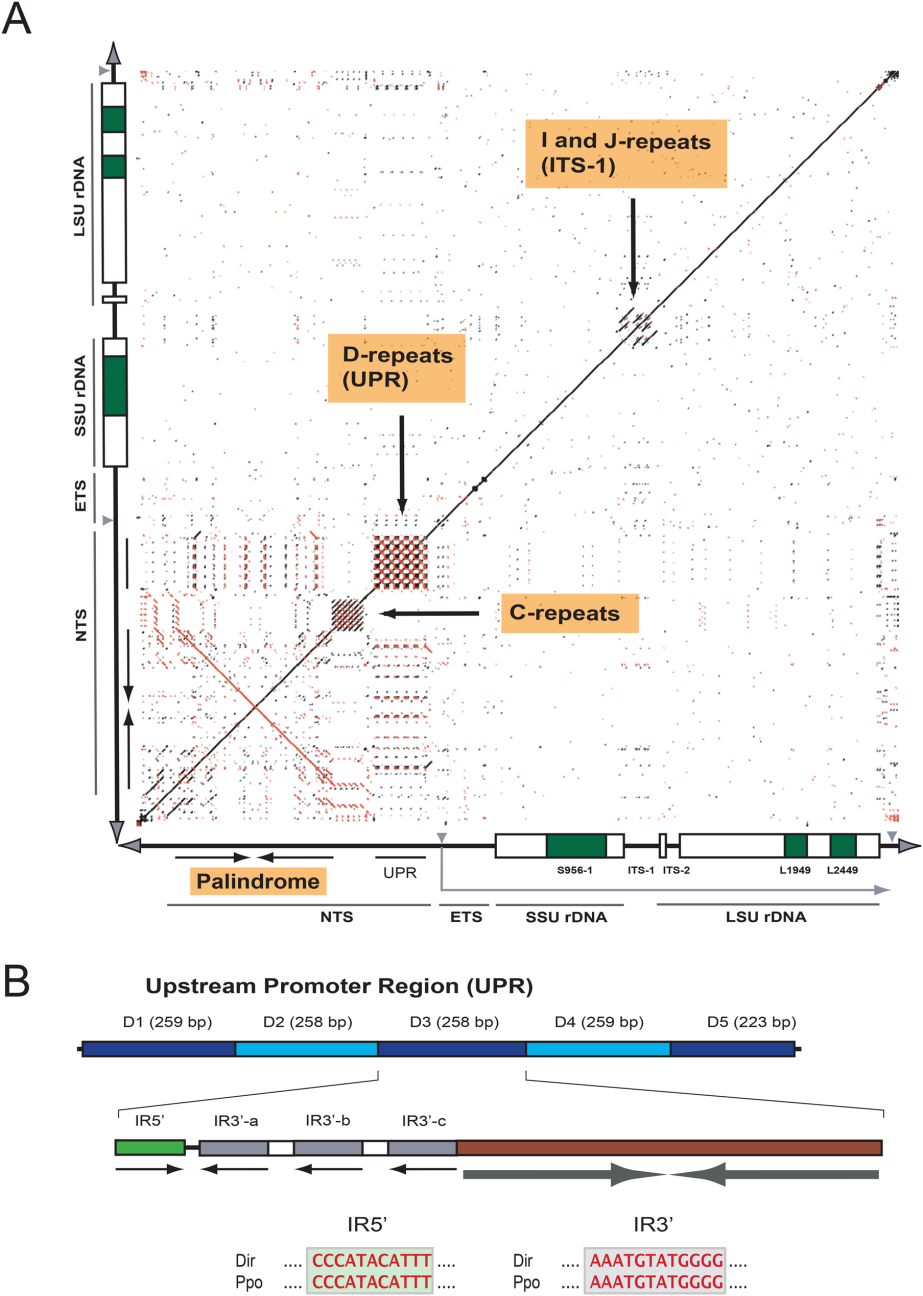
Repetitive features in *D. iridis* rDNA. (**A**) self-identity dot plot of the rDNA chromosome aligned with itself for visualization of complex repeats. Red dots, sense direction; blue dots, reverse directions. Direct repeat motifs C, D, I, and J are indicated (see legend to Fig. 2 for annotation details). (**B**) expended view of the upstream promoter region (UPR). Each of the five copies consist of two inverted repeat (IR) motifs, a 140-bp palindrome and a dynamic IR. The latter consists of one 5’ part (IR5’) and three alternative 3’ parts (IR3’-a to c). Sequence comparison of IR structure sequences in *Didymium* (Dir) rDNA and similar sequences in *Physarum* (ppo) rDNA is shown below

The 20.3 kb *D. iridis* rDNA chromosome was sequenced using high-coverage ONT. The rDNA chromosome harbors a single rRNA gene transcription unit of 11.3 kb (Fig. 2A). We identified a highly complex non-transcribed sequence (NTS) region that includes a palindromic sequence (Supplementary Table S1A). Several smaller direct and inverted repeat motifs, designated A to N, were recognized in the NTS, external transcribed spacer (ETS), internal transcribed spacer 1 (ITS-1), and the downstream sub-telomeric region (Fig. 2; Fig. 3A). Interestingly, sequences of the individual repeat units comprising direct repeat arrays were highly similar but not identical (Supplementary Fig. S1).

### TTAGGG telomeric repeats and non-transcribed sequence region

The left and right telomeric regions contained arrays of CCCTAA and TTAGGG repeats, respectively, as well as sub-telomeric direct repeat motifs (Fig. 2B). The left telomeric repeat extended into a 4.2 kb palindromic region rich in simple sequences and homopolymers (Fig. 3A). The forward and reverse palindrome halves were 99% identical in sequence (Supplementary Fig. S2). The palindrome contained unique motifs that resembled the *P. polycephalum* rDNA replication origins. Remarkably, these regions (designated PRO1 and PRO2) contained 15 bp sequences identical to those in *P. polycephalum* (Fig. 2C), arguing for putative origins of replication in *D. iridis* rDNA. The *P. polycephalum* rDNA replication origins were determined more than four decades ago using a combination of electron microscopy, Maxam-Gilbert DNA sequencing, and various biochemical approaches [12, 21, 22].

The right side of the palindrome was flanked by direct repeat motifs extending into a complex upstream RNA polymerase I promoter region (UPR). The UPR was organized as a direct repeat array consisting of the 260 bp D-type motif (Fig. 2C; Fig. 3B). Different D-type copies were highly similar but not identical to each other (Supplementary Fig. S1). The D motif has a 140 bp palindrome at the right end and a complex arrangement of shorter inverted and direct repeats at the left end (Fig. 3B). The latter involves three alternative inverted repeat structures using one common IR5’ and three variant IR3’s. IR5’ and IR3’ have almost identical counterparts in *P. polycephalum* rDNA [22] (Fig. 3B).

### External and internal transcribed spacers

The ETS was defined as the sequence region between the pre-rRNA transcription start and the 5’ end of the SSU rRNA gene. The transcription start site was determined by sequence similarity to the corresponding site in *P. polycephalum* [46] and by pre-rRNA read mapping in *D. iridis*. The 1,483 bp ETS contained four different direct repeat motif types, designated E to H, in two to five copies (Fig. 2D). Furthermore, ITS-1 harbored I- and J-type direct repeats in three and two copies, respectively (Fig. 2E). The number of individual copies of the J-type motif appeared strain-specific in *D. iridis*. The Pan 2, Pan 3 and CR 8 isolates contained three J-type copies, while the Hon 1 and Gua 1 isolates had four copies [27] (Fig. 2E).

### Processing of the rRNA precursor results in expression of a homing endonuclease gene

**Fig. 4:**
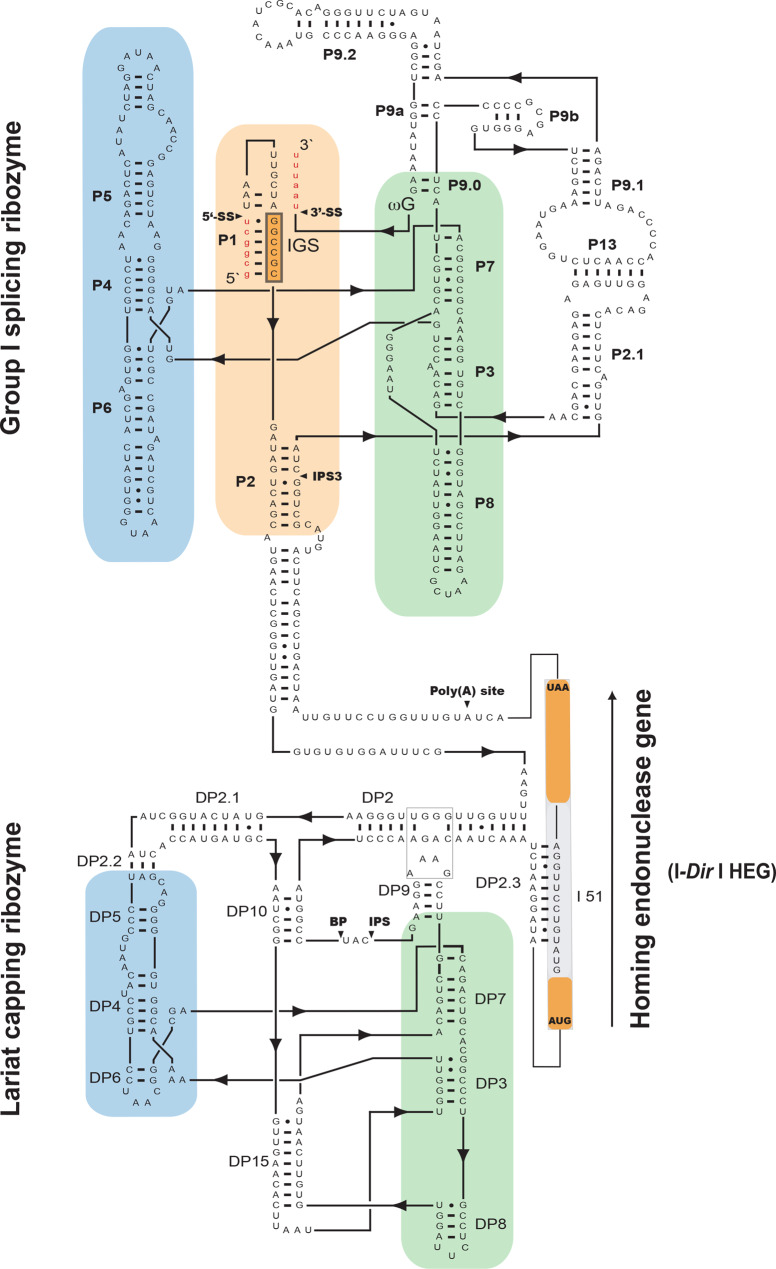
Updated secondary structure diagram of the mobile group I intron S956-1 from *D. iridis*. The intron consists of three structural and functional elements: group I splicing-ribozyme, lariat capping ribozyme, and homing endonuclease gene. The two latter elements are inserted into P2 of the splicing ribozyme. Paired RNA segments are denoted DP2 to DP15 in the lariat capping ribozyme and P1 to P13 in the splicing ribozyme. The ribozyme catalytic domain, folding domain, and substrate domain are indicated by green, blue and orange boxes, respectively. 5’ SS and 3’ SS, splicing sites; IPS and IPS3, internal processing sites; BP, branch site; poly(A) site, polyadenylation site; IGS, internal guide site; I51, 51 nt spliceosomal intron. Ribosomal RNA exon sequences franking the intron are indicated by red lowercase letters in the splicing ribozyme

The SSU rRNA gene encodes a 1916 nt rRNA (19S) and a 1436 nt mobile group I intron named S956-1 (Supplementary Table S1B). In vitro structural features of S956-1 have been reported previously [29, 47]. In brief, the *D. iridis* S956-1 is a typical mobile group I intron with a complex RNA structure, and an updated secondary organization is presented in Fig. 4. The intron harbors two ribozymes with different functions (a group I ribozyme and a lariat-capping ribozyme), and a homing endonuclease gene (HEG). The HEG is further intervened by a small 51-nt spliceosomal intron (designated I51). The S956-1 intron is optional in *D. iridis* and is present only in the Pan 2 isolate. The LSU rRNA gene encodes two rRNA segments (5.8S and 26S) of 154 bp and 3702 bp, respectively. The 26S rRNA segment is further interrupted by the mandatory group I introns L1949 and L2449 (Fig. 2A; Supplementary Table S1B).

**Fig. 5:**
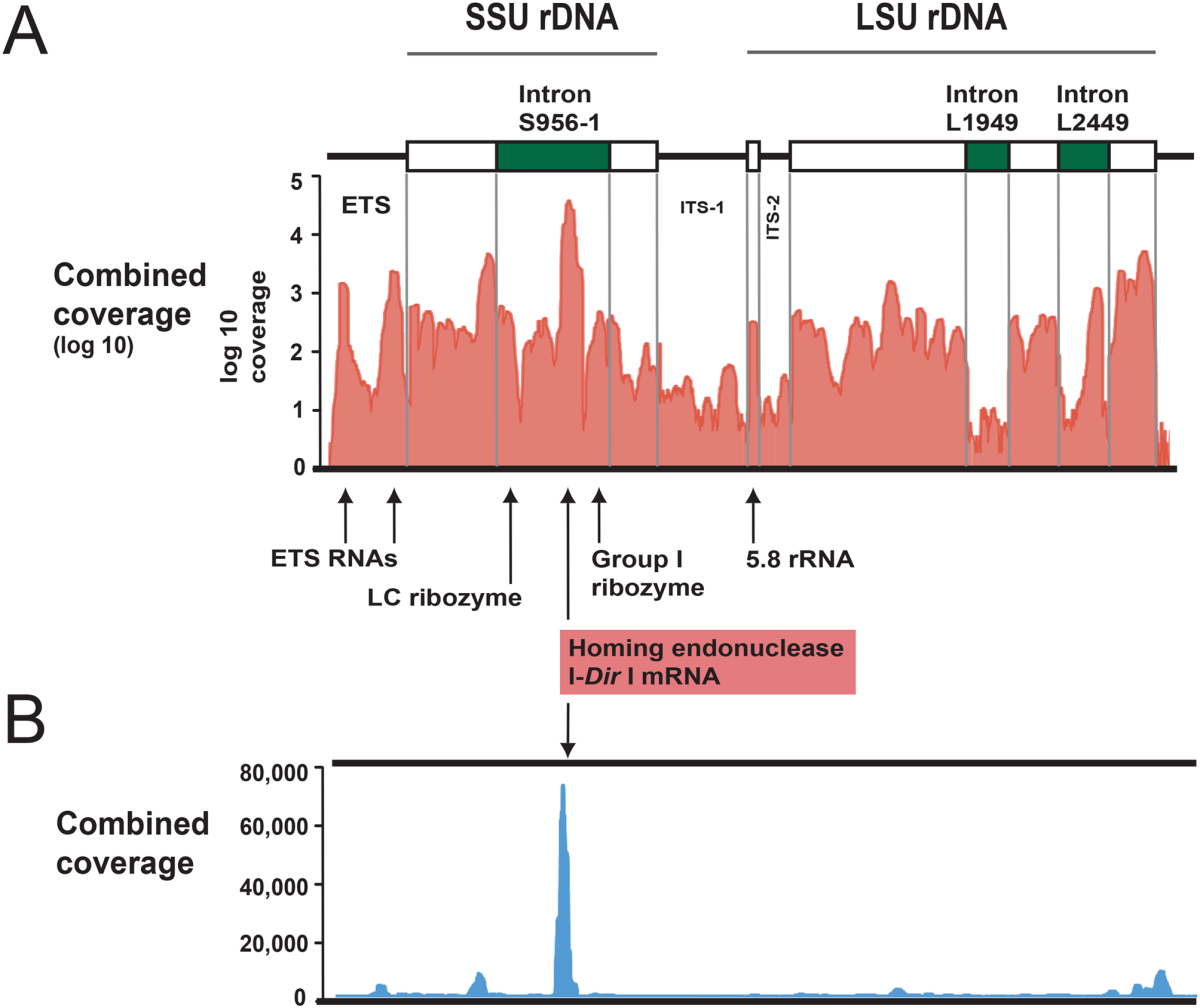
RNA mapping of the RNA polymerase I transcription unit. (**A**) Schematic view of the unit is shown on top, and incudes group I introns (green boxes) and rRNA gene segments (white boxes). Combined Illumina library sequencing reads (amoeba, microcyst, flagellate, and plasmodium) are mapped onto the transcriptional unit. The 3’-end tag libraries have preference for polyadenylated RNA. Note that coverages are displayed as log_10_ values. ETS, external transcribed spacer; ITS, internal transcribed spacer. Homing endonuclease mRNA, 5.8S rRNA, and ribozymes are indicated. No reads map to the non-transcribed spacer region (Supplementary Fig. S3A). RNA read mappings at separate life-stages are shown in Supplementary Figure S3B. (**B**) read map of the combined dataset show that the homing endonuclease mRNA is the dominating polyadenylated transcript

Total RNA was isolated from four *D. iridis* life stages (see Fig. 1) and sequenced. The four stages include three haploid stages (the single-nucleate amoebae, microcyst, and flagellate) and one diploid stage (the multi-nucleate plasmodium). Large numbers of reads mapped to the pre-rRNA transcription unit; we detected no reads mapping to the rest of the rDNA chromosome (Supplementary Fig. S3A). A summary of log_10_ read coverage is presented in Fig. 5A and several interesting features were noted. First, the rRNA exon segments exhibited high coverage. Second, no major differences were observed in the overall mapping patterns between life stages (Supplementary Fig. S3B). Third, ETS reads have a 5’ end that corresponds exactly to the predicted transcription initiation (Ti) start from sequence comparisons to *P. polycephalum* (Fig. 2D), and two enriched ETS RNAs that correspond to the E-type and H-type repeat motifs, respectively, were noted. However, at the current stage we have no indications of their functional role. Fourth, group I introns at positions S956, L1949 and L2449 were all spliced out, and flanking rRNA exon sequences were perfectly ligated in both haploid and diploid stages. Finally, a sub-region of the mobile group I intron S956-1 corresponding to the HEG was enriched at all stages (Supplementary Fig. S3B; Fig. 5B).

**Fig. 6:**
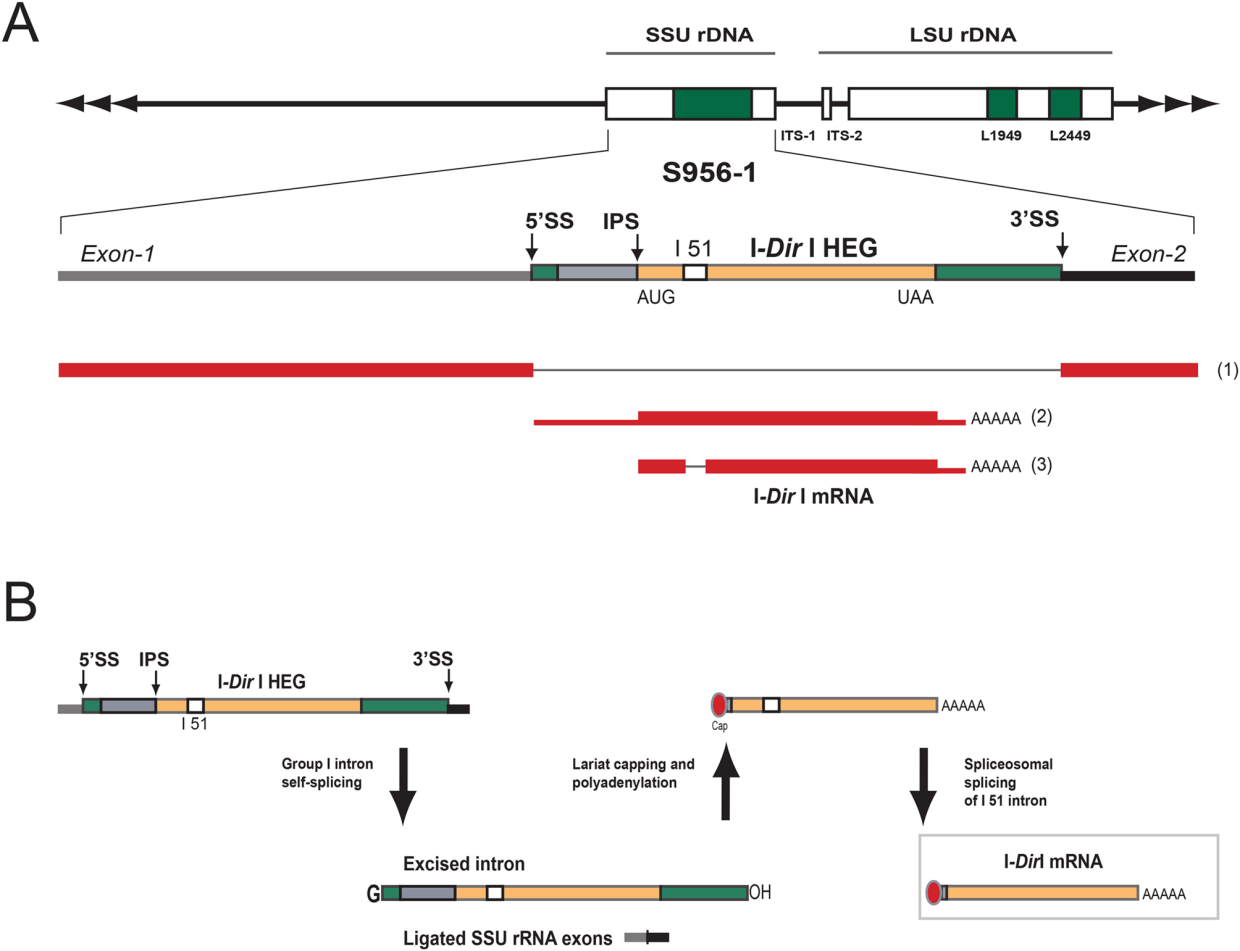
Expression of the intron-encoded homing endonuclease gene (HEG). (A) summary of RNA read mapping results in the SSU rRNA gene region. Three enriched RNA species were detected (RNA1 to 3). Whereas RNA1 corresponds to the spliced SSU rRNA, RNA2 and RNA3 are polyadenylated species covering the intron HEG region. RNA3 represents the I-*Dir* I mRNA and is present in *D. iridis* amoeba, microcyst, flagellate, and plasmodium. (**B**) summary of the expression pathway revealed by previously reported experimental studies. The S956-1 intron is self-spliced from its nucleolar precursor. The HEG region is further processed by adding a lariat cap (red circle) at the 5’ end and a poly(**A**) tail at the 3’ end. Finally, a mature HE mRNA is generated by the removal of a small 51-nt spliceosomal intron. The mRNA is subsequently translocated to the cytoplasm and translated on active ribosomes

The mobile S956-1 intron has been reported to carry RNA processing sites associated with self-splicing, lariat capping modification, and homing endonuclease mRNA maturation (reviewed in [45]). The transcript mapping identified two intron-derived RNA species (RNA2 and RNA3; Fig. 6A; Supplementary Fig. S3B) that were polyadenylated at the same site as previously reported by RT-PCR and Sanger sequencing [33]. While RNA2 corresponded to the unprocessed intron mRNA, RNA3 represented the mature I-*Dir*I mRNA with a perfectly removed I51 intron (Fig. 6B).

## Discussion

We report the telomere-to-telomere sequence of the rDNA chromosome of the myxomycete *D. iridis*. The chromosome is linear in structure, flanked by regular telomeres, and contains a high number of repeat features. Each chromosome carries one rRNA transcription unit that constitutes close to 60% of the rDNA, an RNA polymerase I promoter region, and two putative replication origins. The transcription unit harbors the SSU and LSU rRNA genes, and three group I introns. One of these introns is complex in structure, encodes two ribozymes with different functions, and a homing endonuclease protein. The HEG is further interrupted by a small spliceosomal intron. RNA mapping analysis supports HEG expression in haploid stages, as well as in diploid plasmodia.

The *Didymium* rDNA chromosome contained arrays of regular TTAGGG telomeric repeats at both ends. Several repeats were noted in non-transcribed regions (sub-telomeric regions and NTS) and in transcribed regions (ETS and ITS), features resembling those of the rDNA chromosome in *P. polyceophalum* [21]. An interesting observation is that individual copies of a repeat array are heterogeneous in nature, suggesting sequence fixation during evolution. Similar repeat features appear common in myxomycete rDNA group I introns [28, 48]. The 4.2 kb NTS palindrome carries two putative replication origins that resemble those identified in *P. polycephalum* rDNA [22]. The linear 60 kb rDNA chromosome in *Physarum* is differently organized compared to that of *Didymium*. In *P. polycephalum* the rDNA is found as a giant palindrome containing two diverging rRNA transcription units (reviewed in [11]). Two replication origins have been mapped to each 30 kb palindrome half: one promoter-proximal and one promoter-distal [49]. Only one of the four replication origins is activated in each replication cycle, a process regulated through DNA methylation [23, 50, 51]. Based on sequence feature similarity between *Didymium* and *Physarum*, it is tempting to speculate that DNA methylation also may play a role in *Didymium* rDNA replication.

**Fig. 7:**
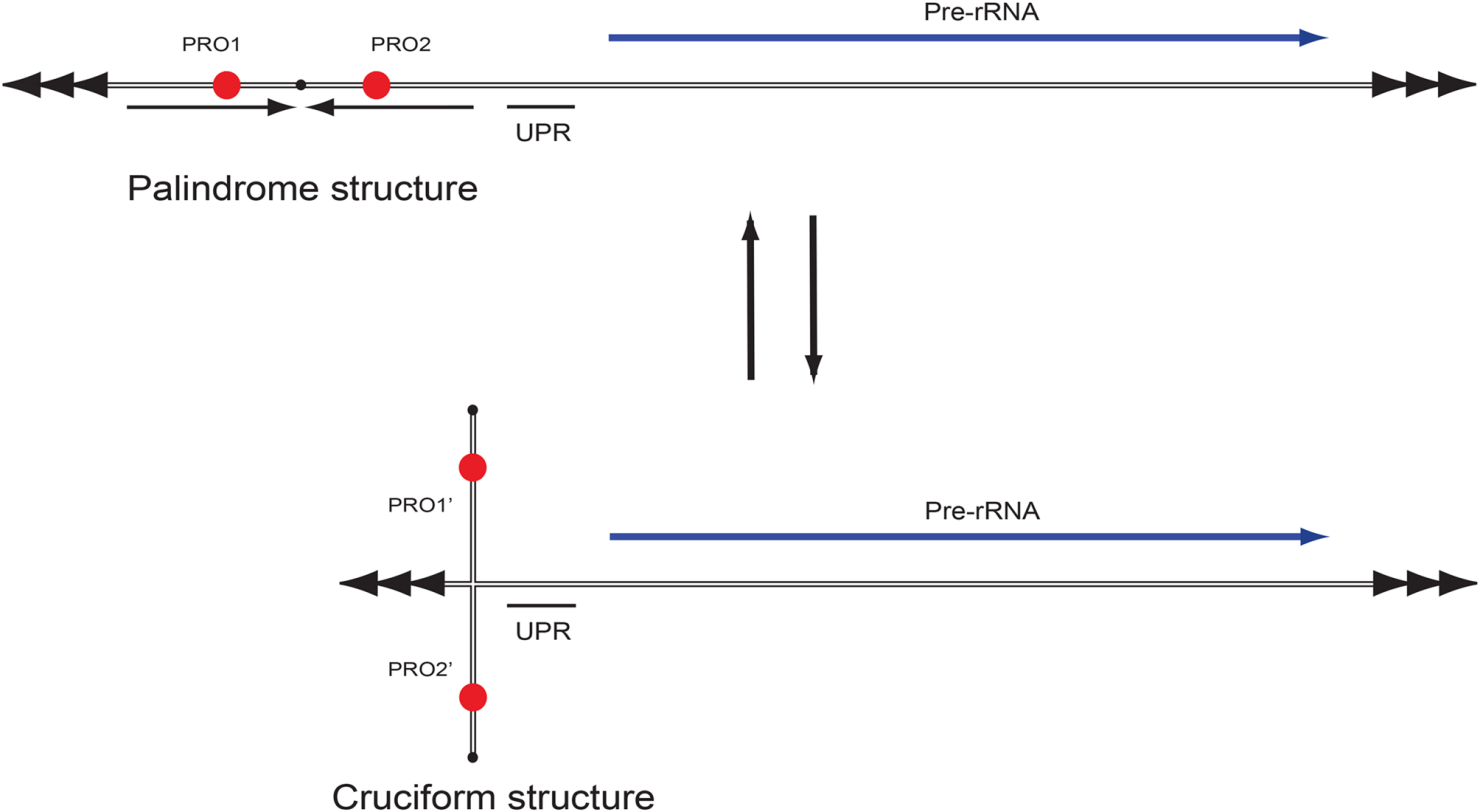
Schematic model of a reversible palindrome to cruciform structure of the rDNA chromosome. We propose four variants of putative DNA replication origins: inter-strand PRO1 and PRO2 in the palindrome structure, and the intra-strand PRO1‘and PRO2’ in the cruciform structure. Telomere sequences are indicated by tandem arrows. UPR, upstream promoter region

DNA cruciform structures are formed by some inverted repeats and are important for many biological processes including DNA replication and transcription initiation [52]. Inverted repeats are particularly common in rDNA, suggesting frequent occurrence of cruciform structures [53]. The *Didymium* rDNA carries a 4.2 kb inverted repeat (palindrome) with almost identical halves (99%). We propose that this palindrome may form a large reversible cruciform switching between inter- and intra-strand base pairing (Fig. 7). Consequently, four different replication origin constellations (PRO1, PRO2, PRO1‘, and PRO2’) could be formed. We hypothesize that strand-specific methylation combined with the formation of DNA cruciform structures may regulate rDNA chromosome replication.

An upstream promoter region (UPR) containing inverted repeats nested within direct repeats, appears to be a conserved feature between *Didymium* and *Physarum* rDNAs. We found that both DNA strands of UTR have the potential to fold into complex secondary structures, including 140-bp cruciform structures and alternative inverted repeat structures. Mapping of total RNA reads to the rDNA chromosome shows that rRNAs were enriched throughout the *Didymium* life cycle, and all group I introns were perfectly spliced out. Of particular interest are the RNA reads that mapped to the HEG region of the mobile intron, which include two polyadenylated RNA species. One of these represents the mature HE mRNA, and our data support HEG expression both in haploid amoebae and flagellates as well as in diploid plasmodia. This implies that the complex group I intron Dir.S956-1 is active as a mobile element in all the vegetative life stages.

## Conclusions

The rDNA locus in eukaryotes is in general highly repetitive in nature and present in hundreds of copies per haploid genome [54]. Additionally, each rDNA copy harbors a plethora of direct and inverted repeat features in the NTS [4]. Repetitive sequences are very challenging to resolve using most DNA sequencing technologies, but recent developments in ONT long-read single-molecule sequencing have greatly improved the resolution of complex sequences [55]. Based on ONT sequencing, the *D. iridis* rDNA revealed a dense composition and variation of repeat features ranging from a 4.2 kb palindrome to microsatellite-like direct repeats. Within and between the repeat features, putative replication origins, an RNA polymerase I transcription start site, and transcription-regulating sequences were identified. Transcriptome mapping analysis showed extensive expression of rRNA throughout the life cycle. Our RNA sequences confirm that group I intron activities previously demonstrated in vitro also occur in haploid and diploid cell stages, strongly arguing for the functional importance of these mobile genetic elements.

## Electronic supplementary material

Below is the link to the electronic supplementary material.

Supplementary Material 1

Supplementary Material 2

Supplementary Material 3

Supplementary Material 4

Supplementary Material 5

## Data Availability

The datasets presented in this study can be found in online repositories under the GenBank accession number PZ013293 and the NCBI BioProject number PRJNA1423643 (BioProject SRA accession numbers SRX32166292, SRX32166293, SRX32166294, SRX32166295, and SRX32166296).
