# Supplementary material for: Telomere-to-telomere characterization of rDNA chromosome in the myxomycete Didymium iridis
Source: BMC Mol Cell Biol. 2026 Apr 6;27:30. doi: 10.1186/s12860-026-00587-7 (PMC13182076; doi:10.1186/s12860-026-00587-7)
Supplement: Supplementary file 1 — Supplementary Material 1 [file 12860_2026_587_MOESM1_ESM.pdf]

Supplementary Figure S1. Inverted and direct repeat motifs in *D. iridis* rDNA. (*) indicates gap in sequence alignments.

A-motif:

```
A1      TGGTCGATTTCTCGCGATGTGTTGGCGGGGTGCCCTAACCCTATATGGACGAAATCACCCTCTTGCGAGCACAAGTACTC 80
A2      TGGTCGATTTCTCGCGATGTGTTGGCGGGGTCCCATAGGTATGTATGGACGAAATTACCCTCTTGCGAGCACAAGTACTC 80

A1      CCATATTTTTTTTAATTTTTTATCTCTCAGTGGGAGCCCAGCAGACACTGTTTCGACCCTAGCCCCAACCGCG*AAAATC 159
A2      CCATATTTTTTTTAGTCCAGTATCTTTCACTGGGAGCTCAGGAGACACTGTCTCGACCTCGATTCCGACCGCGAAAAACC 160

A1      GCGAAAAAACCTCGAAAATCGACCCAAAAACACCTGAAAAAAAAAGTTCGAATCGAGGTCTAGTAAGG 227
A2      GCGAAAAAACCTCGAAAATCGACCCAAAAACACCTGAAAAAAAA*GTGCCAGTCGAGGTCTAGTAAGG 227
```

B-motif:

```
B1      ACCCGCGACGCGAGAACAGGCGTCCCATATTTTTTTCAATTTTTTACG 48
B2      ACCCTCGCTGCAGCAACAGGCGTCCCATATTTTTTTCGATTTTTTATG 48
```

C-motif:

```
C1      TCGCCCGGAGGTCGGAGTCGGTGCGGGATCACCTCCCAGAGGGCCCAGGGGGTGGTTTTCGATCTTACTTTGGTTCGATT 80
C2      TCGCCCGGAGGTCGGAGTCGGTGCGGGATTACCTCCCAGAGGGTCCAGGAGGTGGTTTTCGACCTTACTTTGGTTCGATT 80
C3      TCGCCCGGAGGTCGGAGTCGGTGCGGGATTACCTCCTAGAGGGCCCAGGAGGTGGTTTTCGATCTTACTTTGGTTCGATT 80
C4      TCGCCCAGAGGTCGGAGTCGGTGCGGGATTACCTCCTAGAGGGCCCAGGAGGTGGTTTTCGACCTTACTTTGGTTCGATT 80
C5      TCGCCCGGAGGTCGGAGTCGGTGCGGGATTACCTCCTAGAGGGTCCAGGAGGTGGTTTTCGATCTTACTTTGGTTCGATT 80
C6’     TCGCCCAGAGGTCGGAGTCGGTTCCAAATCACCTCCTAGAG 41

C1      CCGACTTCGAAATTCCGACCCCCTGTCGACTTAGCCTAGGGTTAGCGTTGGAGGTCGAAATTTCGAAG 148
C2      CCGACTTCGAAATTCCGACCCCCGGTCAACTGACCCTAGGGTTAGCGTTGGAGGTCGGAATTTCGAAG 148
C3      CCGACTTCGAAATTTTGACCTCCAGTCAACTGACCCTAGGGTTAGCGGTAGGGGTCGAAATTTCGAAG 148
C4      CCGACTTCGAAATTCCGACCCCCGGTCAACTGACCCTAGGGTTAGCGTTGGAGGTCGAAAATCGATAG 148
C5      CCGACTTCGAAATTTTGACCCCCGGTCAACTGACCCTAGGGTTAGCGTTGGAGGTCAAAAATCGATAG 148
```

D-motif:

```
D1      TCCCATACATTTGAAAAACTTAGTTTTTTTTTGAAATGTATGGGGGATTTTGACTTAGTTTTTTTTTGAAAATGTATGGG 80
D2      TCCCATACATTTGAAAAACTTAGTTTTTTTTTGAAATGTATGGGGGATTTTGACTTAGTTTTTTTTTGAAAATGTATGGG 80
D3      TCCCATACATTTGAAAAACTTAGTTTTTTTTTGAAATGTATGGGGGATTTTGACTTAGTTTTTTTTTGAAAATGTATGGG 80
D4      TCCCATACATTTGAAAAACTTAGTTTTTTTTTGAAATGTATGGGGGATTTTGACTTAGTTTTTTTTTGAAAATGTATGGG 80
D5’     TCCCATACATTTGAAAAACTTAGTTTTTTTTTGAAATGTATGGGGGATTTTGACTTAGTTTTTTTTTGAAAATGTATGGG 80

D1      GGAATTTGACTTAGTTTTTTTTTTTGAAATGTATGGGAGGTCCCCCC***GGTCTCGGACCCCCCCGACTTGGGTCGACCC 157
D2      GGAATTTGACTTAGTTTTTTTTTTTGAAATGTATGG*AGGTCTCCCC***GGTCTCGGACTCCC**GACTTGGGTCGACCC 154
D3      GGAATTTGACTTAGTTTTTTTTTTTGAAATGTATGGGAGGTCCCCCC***GGTCTCGGACCCCC**GACTTGGGTCGACCC 155
D4      GGAATTTGACTTAGTTTTTTTTTTTGAAATGTATGGGAGGTCCCCCC***GGTCTCGGACTCCCC*GACTTGGGTCGACCC 156
D5’     GGAATTTGACTTAGTTTTTTTTTTTGAAATGTATGGGAGGTCCCCCCCCCGGTCTCGGACCCCC**GACTTGGGTCGACCC 158

D1      GAAGCGCAAATCCCGTTCGAAATCGACCAAAAATTTTTGGTCCGTTTTGAACGGGATTTGCGCTTTGGGTCGACCCAAGT 237
D2      GAAGCGCAAATCCCGTTCGAAATCGACCAAAAATTTTTGGTCCGTTTTGAACGGGATTTGCGCTTTGGGTCGACCCAAGT 234
D3      GAAGCGCAAATCCCGTTCGAAATCGACCAAAAATTTTTGGTCCGTTTTGAACGGGATTTGCGCTTTGGGTCGACCCAAGT 235
D4      GAAGCGCAAATCCCGTTCGAAATCGACCAAAAATTTTTGGTCCGTTTTGAACGGGATTTGCGCTTTGGGTCGACCCAAGT 236
D5’     AAAGCGCAAATCCCGTTCGAAATCGACCAAAAATTTTTGGTCCGTTTTGAACGGGATTTGCGCTT 223

D1      CGGGGGGTCCGAGACC*GGTCCC 259
D2      CGGGGGGTCCGAGACCGGGTCCC 257
D3      CGGGGGGTCCGAGACCGGGTCCC 258
D4      CGGGGGGTCCGAGACCGGGTCCC 259
```

E-motif:

```
E1      TGCCCCCC***GGGGGGGTCTATA***GGGGGGATTTTTT*CAAAATTTTTTTGG 48
E2      TGCCCCCCGGTGGGGGGGTCTAGGGGAGGGGGGATTTTTTTCAAAATTTTTTTGG 55
```

F-motif:

```
F1      AATGG*GATCGGAATCGGGATAGG 23
F2      AATCGTAATAGGAATCGTAATAGG 24
F3      GATCATAATAGGGATCATAATAGG 24
F4      GATCGTAATAGGGATTGTAATAGG 24
F5      AATCGTGATAGGGATCGTAATAGG 24
```

G-motif:

```
           >>>>> loop <<<<<
G1      GGTGGGGG GCAA CCCCCA 18
G2      GGTGGGGG GCAA CCCCCT 18
```

H-motif:

```
H1      GGAC*TGAAGCTGAAGC*CGAAACCGA 25
H2      ACAA*TGAGACTGAAAAATGAGACTGA 26
H3      AAAA*TGAGACCGAAAAATGAGACTGA 26
H4      AAAA*TGAGACCGAAAAATGAGACCGA 26
H5      AAACACGAAACCGAAAC*CGAAACTGA 26
```

I-motif:

```
I1      AAAGACCATGGTGACGGCCTCGCCCTCGGGAGGGGTCCGACCTGTGGCAAATGGGCGGCAAATACGCTGGGGGGAGGGGG 80
I2      AAAGACCACGGTGACGGCCTCCCCCTCGGG*GGGGTCCGACCTGTGGCAATTGGGCGGCAAATACGCTGGGGGGAGGGGG 79
J3      AAAGACCATGGTGACGGCCTCTCCCTCGGG*GGGGTCCGACCTGTGGCAATTGGGCGGCAAATACGCTGGGGGGAGGGGG 79

I1      TGAGACGTCACTTTTTCCCTTTGGCACCACGTCTTGTAACCTCGGTGAATCGGGGGCACTGCTGGGGGGGCGGGCAAGTA 160
I2      CAATTTATTGCTTTTTACCTTTGGC***ATGCCTTGTAACCTCGGTGAACCGGGGGCACTGCTGGGGGGGCGGGCAAGTA 156
J3      CAATTTATTGCTTTTTACCTTTGGC***ACGCCTTGTAACCTCGGTGAACCGGGGGCACTGCTGGGGGGGCGGGCAAGTA 156

I1      GGCCTAACAGCTAAACGACCGCAAAATCCCTTTGGTATGCCTTCAAGATCCTTGGCCAAGGGGC 224
I2      GGCCTAACAGCTAAACGACCGCAAAATCCCTTTGGTACGCCTTCAGGATCCTTGGCCAAGGGGC 220
J3      GGCTTAACAGCTAAACGACCGCAAAATCCCTTTGGTATGCCTTCAGGATCCTTGGCCAAGGGGC 220
```

J-motif:

```
J1      AACATCGGTTCTGGGTTGGGGGGAGACGCCGTCAGGCCGACCTTCCTGGCTTGGGGCAACTGGAAAAGACCTACTGGGAG 80
J2      AACATCGGTTCTGGGTTGGGGGGAGACGCCGTAAGGCCGACCCTTCTGGCTTGGGACAACTGGAAAAGTCCTACTGGGAG 80

J1      TAACATCCTTCACCTTCACTGGCGAGGGCGGGCACTCAAGGGCA***TTTCCGTA**GGTGAACCTGCGGGTGGGTGGC 154
J2      TAACAACCTTCACCTTCACCGGCGAGGGCGGGCACTCAAGGGCACATTATCCGCACTGGTGAACCTGCGGGTGGGCGGC 159
```

K-motif:

```
K1      ACCCCCCCCCTTTTTTTTTCAAAATTTTGAAAATGTAGGGTGGGGTTAAGCGCGAATTACGGT 62
K2      ACACCCCCCCTTTTTTTTTCAAAATTTTGAAAATGTAGGGTGGGGCTAAGCGCAAATTACGGT 62
```

L-motif: **Transcription termination inverted repeat**

```
        >>>>>>>>>>>>>>>    loop   <<<<<<<<<<<<<<<
L       AAAACCCCAATCGAC TTTTTACG GTCGATTGGGGTTTT 38
```

M-motif:

```
M1      GGGGGAGGACAGCGATTTTTTTAGGGTTACGGCGTGATGG 40
M2      GGAGGGGGACAGCGATTTTTTTAGGGTTAGCGGCGGATGG 40
```

N-motif:

```
N1      TTAGGGTTACGA****GACCTAAATTTGCGCCCTTTTTCGAAGCAAAAGTTGGGGTTAGGGGGCGTAAAATCAGGTCAAA 76
N2      TTAGGGTTAGAGGGA*GACCTGAAATTGCGCTCTTGGTCGAGGCAAAAGTTGGGGTTAGGGGGCGTAAAATCAGGTCAAA 79
N3      TTAGGGTTAGGGTTACGACCGAAAAATGCGCTCTTGGTCGAGGTGAAAATCGAAAACAGGGGGCGTAAAATCAGGTCAAA 80
```
