# Supplementary material for: Telomere-to-telomere characterization of rDNA chromosome in the myxomycete Didymium iridis
Source: BMC Mol Cell Biol. 2026 Apr 6;27:30. doi: 10.1186/s12860-026-00587-7 (PMC13182076; doi:10.1186/s12860-026-00587-7)
Supplement: Supplementary file 2 — Supplementary Material 2 [file 12860_2026_587_MOESM2_ESM.pdf]

Supplementary Figure S2. A comparison of forward and reverse sequences in non-transcribed spacer palindrome. The alignment was generated in BLAST and revealed 99% identical positions. Dashes indicate deleted positions, and vertical lines indicate identical positions.

Forward: 2094 bp; Reverse: 2094 bp; Identity: 98.85%; PRO (putative replication origin) -1 and 2: Green marks

```
Forward 1    AGTAAGGTCGAAAACCATCCCCAGGGCCCTCCTGGAGGTCATTTGGAACCGACTCCGACC  60
             |||||| |||||||||| |||| ||||||||  |||||| ||   | |||||||||||||
Reverse 1    AGTAAGATCGAAAACCACCCCCTGGGCCCTCTGGGAGGTGATCCCGCACCGACTCCGACC  60

Forward 61   TCCGGGAGACGAttttttttCGACGaaaaaaaCCGAGAAAACCGGGGTCCCCACGCGTT  120
             |||||| || ||||||||||||||||||||||||||||||| | ||||| ||||||||||
Reverse 61   TCCGGGCGATGATTTTTTTTCGACGAAAAAAAACCGAGAAAA-CAGGGGTACCCACGCGTT  119

Forward 121  TTTTCGAGAAAAACCGGTTTCGTATCTCGCGAATTTGATCGCCCGACGTAGCCACGTG  180
             ||||||||      ||||||||||||||||||||||||||||||||||||||||||||||
Reverse 120  TTTTCGAG-GGGGGCGGTTTCGTATCTCGCGAATTTGATCGCCCGACGTAGCCACGTG  178

Forward 181  GGACATTTTTCACCCTCGCTGCAGCAACAGGCGTCCCATAtttttttcgatttttttATGC  240
             ||||||||||||||||||||||||||||||||||||||||||||||||||||||||||||
Reverse 179  GGACATTTTTCACCCTCGCTGCAGCAACAGGCGTCCCATATTTTTTTCGATTTTTTTATGC  238

Forward 241  CAGCGCTCCCCCGAGATGCCGAAACGGACGGCGGTTCCCGCGAACAAAATCGAAAAAACC  300
             ||||||||||||||||||||||||||||||||||||||||||||||||||||||||||||
Reverse 239  CAGCGCTCCCCCGAGATGCCGAAACGGACGGCGGTTCCCGCGAACAAAATCGAAAAAACC  298

Forward 301  ATGGTTTTCGCGCGTTTTTTCTCGAAAAATCGCGTTCGTATCTCGCACGGATTTGGTCCC  360
             ||||||||||||||||||||||||||||||||||||||||||||||||||||||||||||
Reverse 299  ATGGTTTTCGCGCGTTTTTTCTCGAAAAATCGCGTTCGTATCTCGCACGGATTTGGTCCC  358

Forward 361  CTGACGTAGCCCCATAGGACATTTTTCACCCGCGACGCACCAATAGGCTTCCCATAtttt  420
             ||||||||||||||||||||||||||||||||||||||||||||||||||||||||||||
Reverse 359  CTGACGTAGCCCCATAGGACATTTTTCACCCGCGACGCACCAATAGGCTTCCCATATTTT  418

Forward 421  tttcaatttttttGTGGTGGCGTTCTATGGGTGATACCAAAATGTATGGGGGCCCCATACA  480
             ||||||||||||||||||||||||||||||||||||||||||||||||||||||||||||
Reverse 419  TTTCAATTTTTTTGTGGTGGCGTTCTATGGGTGATACCAAAATGTATGGGGGCCCCATACA  478

Forward 481  TTTTTCGGAAAAAAATTCACAAAGTTTTTCGAAATGTATGGAAAATGATTTTGACTCGGGT  540
             ||||||||||||||||||||||||||||||||||||||||||||||||||||||||||||
Reverse 479  TTTTTCGGAAAAAAATTCACAAAGTTTTTCGAAATGTATGGAAAATGATTTTGACTCGGGT  538

Forward 541  CACGACCGAAAAATGCGGACCAAAACCTCGGGCGCGAGCTGCGGCAGGGGTAAAAATCAG  600
             ||||||||||||||||||||||||||||||||||||||||||||||||||||||||||||
Reverse 539  CACGACCGAAAAATGCGGACCAAAACCTCGGGCGCGAGCTGCGGCAGGGGTAAAAATCAG  598

Forward 601  GTCAAAAAAATTTCGCGCGGCGGCGCAAAAATTCCTGGGAACGCCCCCCAAAAAACCGGAT  660
             ||||||||||||||||||||||||||||||||||||||||||||||||||||||||||||
Reverse 599  GTCAAAAAAATTTCGCGCGGCGGCGCAAAAATTCCTGGGAACGCCCCCCAAAAAACCGGAT  658
```

```
Forward 661  TTTTGACCCCCTTTTTATCGACGAATTGGCCCTTTGGTGGGTCGAAAAATGGTCCTGGCA  720
             ||||||||||||||||||||||||||||||||||||||||||||||||||||||||||||
Reverse 659  TTTTGACCCCCTTTTTATCGACGAATTGGCCCTTTGGTGGGTCGAAAAATGGTCCTGGCA  718

Forward 721  CCGACTCCGGGGTGCATCCGACGTTCTGGCGCCGATATCGATGTATGGGGGTTTTAGTGC  780
             ||||||||||||||||||||||||||||||||||||||||||||||||||||||||||||
Reverse 719  CCGACTCCGGGGTGCATCCGACGTTCTGGCGCCGATATCGATGTATGGGGGTTTTAGTGC  778

Forward 781  GAAAAAGTCGAAAAAGTCGACTTTTTCGAAAAGTGTCACCGTGTATTTTGTATGGGGCGA  840
             ||||||||||||||||||||||||||||||||||||||||||||||||||||||||||||
Reverse 779  GAAAAAGTCGAAAAAGTCGACTTTTTCGAAAAGTGTCACCGTGTATTTTGTATGGGGCGA  838

Forward 841  GGTCGTATCTCGCACGGATTCGCTCCCCAGAGGTAGCACCGTGGACCATTTTTCACTGTG  900
             ||||||||||||||||||||||||||||||||||||||||||||||||||||||||||||
Reverse 839  GGTCGTATCTCGCACGGATTCGCTCCCCAGAGGTAGCACCGTGGACCATTTTTCACTGTG  898

Forward 901  CTCGCACGAATAGGTGCGCCATAtttttttcaatttttttATGACCGTGTTTTACGGGTGA  960
             ||||||||||||||||||||||||||||||||||||||||||||||||||||||||||||
Reverse 899  CTCGCACGAATAGGTGCGCCATATTTTTTTCAATTTTTTTATGACCGTGTTTTACGGGTGA  958

Forward 961  TATTGCTggggggt-gggggtaggt-gggggggggggTCCTCCCATACaaaaaaaTGTAT  1018
             |||||||||||||| |||||||||| ||||||||||||||||||||||||||||||||||
Reverse 959  TATTGCTGGGGGGTGGGGGGTAGGTGGGGGGGGGGGGTCCTCCCATACAAAAAAATGTAT  1018

Forward 1019 GGAGTTTTGATTTCGAAGTCGAGTTGATCCGTATTTTTCGGTCGGTCGCGATCAGAGTTT  1078
             ||||||||||||||||||||||||||||||||||||||||||||||||||||||||||||
Reverse 1019 GGAGTTTTGATTTCGAAGTCGAGTTGATCCGTATTTTTCGGTCGGTCGCGATCAGAGTTT  1078

Forward 1079 TCAAAAAATGGACCAATTCGGGCTTTATAGCCTACGACTTTTCTGACCTCGAATCACAAC  1138
             ||||||||||||||||||||||||||||||||||||||||||||||||||||||||||||
Reverse 1079 TCAAAAAATGGACCAATTCGGGCTTTATAGCCTACGACTTTTCTGACCTCGAATCACAAC  1138

Forward 1139 CTCAGACCATATATGCTAATATTCTATGACGCATACCCTAAACAGCGTTGCATGGTCCAA  1198
             ||||||||||||||||||||||||||||||||||||||||||||||||||||||||||||
Reverse 1139 CTCAGACCATATATGCTAATATTCTATGACGCATACCCTAAACAGCGTTGCATGGTCCAA  1198

Forward 1199 CTCGCGTTCCTCTAGCATCAAAGGTGGAGTTTTTAGAAACCTGAAATCCGTGATTTTGGC  1258
             ||||||||||||||||||||||||||||||||||||||||||||||||||||||||||||
Reverse 1199 CTCGCGTTCCTCTAGCATCAAAGGTGGAGTTTTTAGAAACCTGAAATCCGTGATTTTGGC  1258

Forward 1259 tttttttCGGGTCCAAAATCCTATGGTCCACCCATACATTTTGTATGGAAACCCGAAAAT  1318
             ||||||||||||||||||||||||||||||||||||||||||||||||||||||||||||
Reverse 1259 TTTTTTTCGGGTCCAAAATCCTATGGTCCACCCATACATTTTGTATGGAAACCCGAAAAT  1318

Forward 1319 GGTACCCTCAGAGTACGTATCTCCGTAACGCCTAATTACCCAGCTATGAGGCCTGGGTAG  1378
             ||||||||||||||||||||||||||||||||||||||||||||||||||||||||||||
Reverse 1319 GGTACCCTCAGAGTACGTATCTCCGTAACGCCTAATTACCCAGCTATGAGGCCTGGGTAG  1378
```

```
Forward 1379  AATACTCAACTTTCGAGTGTAAGCATCTATTATATTACCATATAAAATATAATTAAGATT  1438
              ||||||||||||||||||||||||||||||||||||||||||||||||||||||||||||
Reverse 1379  AATACTCAACTTTCGAGTGTAAGCATCTATTATATTACCATATAAAATATAATTAAGATT  1438

Forward 1439  TCACGGAGATACGCCATAGAGCTGTATCTCTGCATGTGTGAGTTCAAATCAGCTATGCGT  1498
              ||||||||||||||||||||||||||||||||||||||||||||||||||||||||||||
Reverse 1439  TCACGGAGATACGCCATAGAGCTGTATCTCTGCATGTGTGAGTTCAAATCAGCTATGCGT  1498

Forward 1499  CAGGATGCAAGCAGATGGCATTCACGCCCACAACATACGTTTCAGGTTCCAAAATCCGAA  1558
              ||||||||||||||||||||||||||||||||||||||||||||||||||||||||||||
Reverse 1499  CAGGATGCAAGCAGATGGCATTCACGCCCACAACATACGTTTCAGGTTCCAAAATCCGAA  1558

Forward 1559  TACTACATAAAAAGATATGAGGTCAAAATCGTTACTTTTGCCGATACGACACCTCTGAAT  1618
              ||||||||||||||||||||||||||||||||||||||||||||||||||||||||||||
Reverse 1559  TACTACATAAAAAGATATGAGGTCAAAATCGTTACTTTTGCCGATACGACACCTCTGAAT  1618

Forward 1619  TGGCACGCGTCATGCATAAATACAACAAAAAATCGCTGCCAAGCCAGAGTTGTCGCATCT  1678
              ||||||||||||||||||||||||||||||||||||||||||||||||||||||||||||
Reverse 1619  TGGCACGCGTCATGCATAAATACAACAAAAAATCGCTGCCAAGCCAGAGTTGTCGCATCT  1678

Forward 1679  CACTTTTTTACCATACATTTTCATATCTCGCCAACGGATGATTTTCAGGACCTGAGGTGT  1738
              ||||||||||||||||||||||||||||||||||||||||||||||||||||||||||||
Reverse 1679  CACTTTTTTACCATACATTTTCATATCTCGCCAACGGATGATTTTCAGGACCTGAGGTGT  1738

Forward 1739  TTAAAATCGTTCTCAACGCACGAAACTGGGGTTATATAGTGACCTGTGAttttttttaca  1798
              ||||||||||||||||||||||||||||||||||||||||||||||||||||||||||||
Reverse 1739  TTAAAATCGTTCTCAACGCACGAAACTGGGGTTATATAGTGACCTGTGATTTTTTTTACA  1798

Forward 1799  ttttttttAGGGGGGTCATTTTGTATGGGCTCCCATACATTTGAAAAACTTAGttttttt  1858
              ||||||||||||||||||||||||||||||||||||||||||||||||||||||||||||
Reverse 1799  TTTTTTTTAGGGGGGTCATTTTGTATGGGCTCCCATACATTTGAAAAACTTAGTTTTTTT  1858

Forward 1859  ttGAAATCGAAAAGTCAAAAATGGGTCCCTACGCTACCGACAGTCCCGACTTTTTTCCAA  1918
              ||||||||||||||||||||||||||||||||||||||||||||||||||||||||||||
Reverse 1859  TTGAAATCGAAAAGTCAAAAATGGGTCCCTACGCTACCGACAGTCCCGACTTTTTTCCAA  1918

Forward 1919  AACCCGAACACGTCCCGCCCAAAACGGACCTGCCGaaaaaaaGTCGCTACAGGGCCCCCG  1978
              ||||||||||||||||||||||||||||||||||||||||||||||||||||||||||||
Reverse 1919  AACCCGAACACGTCCCGCCCAAAACGGACCTGCCGAAAAAAAGTCGCTACAGGGCCCCCG  1978

Forward 1979  GAGGTCCCCAGAATTTTACCAGGACATATTTTTGGCCTAAAATCACGTAAAAAACACGAT  2038
              ||||||||||||||||||||||||||||||||||||||||||||||||||||||||||||
Reverse 1979  GAGGTCCCCAGAATTTTACCAGGACATATTTTTGGCCTAAAATCACGTAAAAAACACGAT  2038

Forward 2039  TTTGAACCTATCTCATCTCGATTTTGCGATCCAACGAAGATACAGAGAGGGGTCTC  2094
              ||||||||||||||| ||||||||||||||||||||||||||||||||||||||||
Reverse 2039  TTTGAACCTATCTCACCTCGATTTTGCGATCCAACGAAGATACAGAGAGGGGTCTC  2094
```
