# Supplementary figures and images for: Telomere-to-telomere characterization of rDNA chromosome in the myxomycete Didymium iridis
Source: BMC Mol Cell Biol. 2026 Apr 6;27:30. doi: 10.1186/s12860-026-00587-7 (PMC13182076; doi:10.1186/s12860-026-00587-7)

A

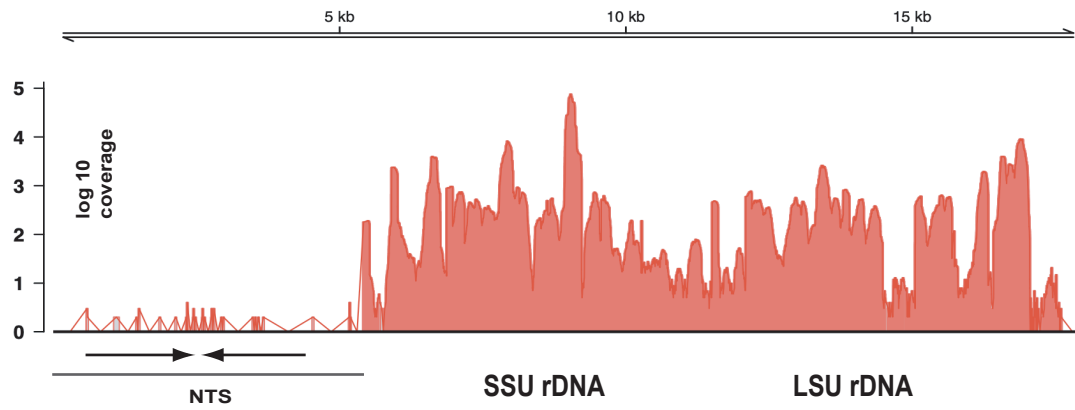

B

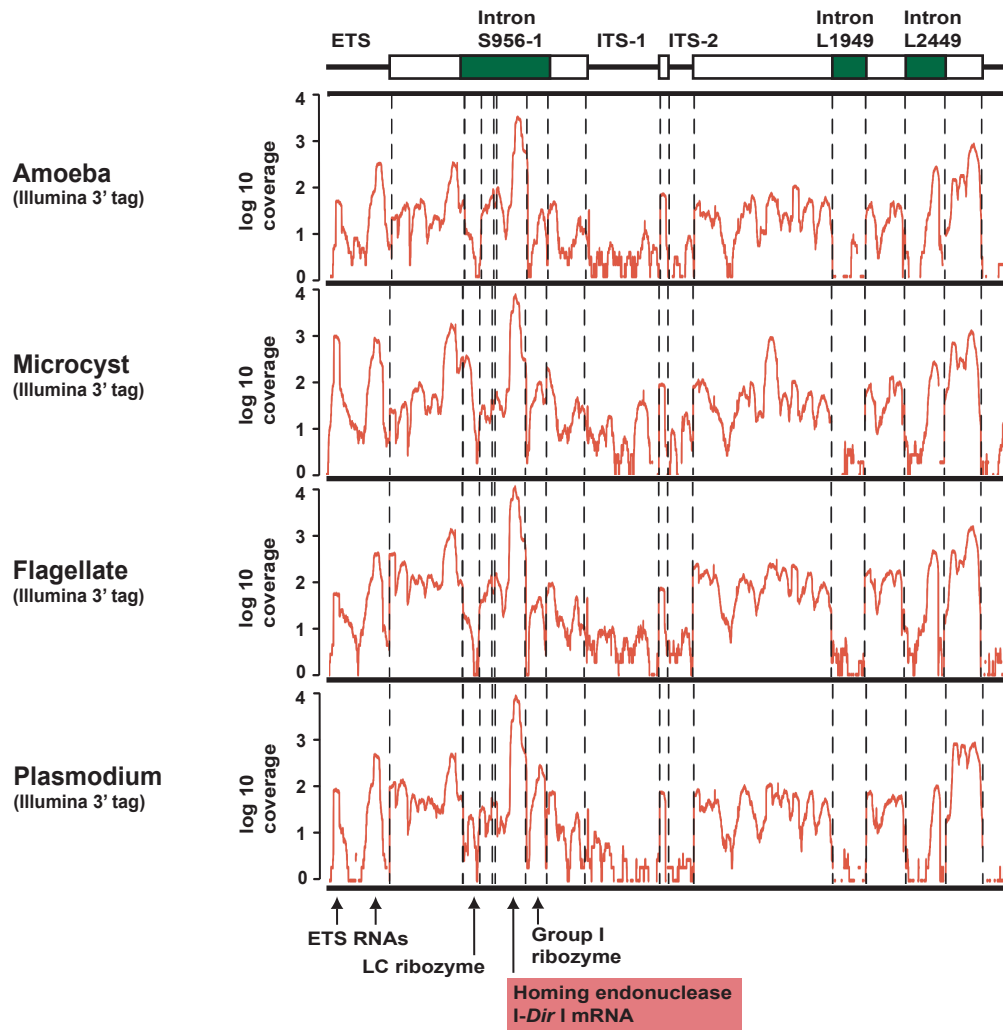

Figure S3

Supplement: Supplementary file 3 — Supplementary Material 3 [file 12860_2026_587_MOESM3_ESM.pdf]
