# Supplementary material for: Telomere-to-telomere characterization of rDNA chromosome in the myxomycete Didymium iridis
Source: BMC Mol Cell Biol. 2026 Apr 6;27:30. doi: 10.1186/s12860-026-00587-7 (PMC13182076; doi:10.1186/s12860-026-00587-7)
Supplement: Supplementary file 4 — Supplementary Material 4 [file 12860_2026_587_MOESM4_ESM.pdf]

Additional file: Supplementary Table S1.
Annotation of *D. iridis* (Pan 2-16) ribosomal DNA chromosome (PZ013293).

A) Repeat features

Feature		Position	Size (bp)
Left telomeric repeat		1 – 300	300
Direct repeat	A1	417 – 643	227
	A2	803 – 1029	227
Direct repeat	B1	644 – 691	48
	B2	1214 – 1261	48
Palindrome	Forward	1023 – 3116	2094
	Reverse	3125 – 5218	2094
Direct repeat	C1	5150 – 5297	148
	C2	5298 – 5445	148
	C3	5446 – 5593	148
	C4	5594 – 5741	148
	C5	5742 – 5889	148
	C6′	5890 – 5930	41
Direct repeat	D1	6264 – 6521	258
	D2	6522 – 6778	257
	D3	6779 – 7036	258
	D4	7037 – 7295	259
	D5′	7296 – 7518	223
Direct repeat	E1	7917 – 7964	48
	E2	8033 – 8087	55
Direct repeat	F1	8847 – 8869	23
	F2	8870 – 8893	24
	F3	8894 – 8917	24
	F4	8918 – 8941	24
	F5	8942 –8965	24
Inverted repeat	G1	9027 – 9044	18
	G2	9045 – 9062	18
Direct repeat	H1	9092 – 9116	25
	H2	9117 – 9142	26
	H3	9143 – 9168	26
	H4	9169 – 9194	26
	H5	9195 – 9220	26
Direct repeat	I1	12724 – 12947	224
	I2	13105 – 13324	220
	I2	13332 – 13551	220
Direct repeat	J1	12948 – 13101	154
	J2	13552 – 13710	159
Direct repeat	K1	19501 – 19562	62
	K2	19565 – 19626	62
Inverted repeat	L1	19646 – 19684	18
Direct repeat	M1	19710 – 19749	40
	M2	19750 – 19789	40
Direct repeat	N1	19810 – 19885	76
	N2	19886 – 19964	79
	N3	19965 – 20044	80
Right telomeric repeat		20045 – 20344	300

B) Gene features

Feature	Position	Size (bp)
External transcribed spacer	7796 – 9319	1524
Small subunit ribosomal RNA gene	9320 – 12671	3352
Exon1	9320 – 10581	1262
Group I intron S956-1	10582 – 12017	1436
Exon2	12018 – 12671	654
Homing endonuclease gene	10869 – 12017	786
Exon1	12018 – 11082	214
Spliceosomal intron I51	11083 – 11133	51
Exon2	11134 – 11654	521
Internal transcribed spacer 1	12672 – 13942	1271
5.8S ribosomal RNA gene	13943 – 14096	154
Internal transcribed spacer 2	14097 – 14527	431
Large subunit ribosomal RNA gene	14528 – 19496	4969
Exon1	14528 – 16913	2386
Group I intron L1949	16914 – 17487	574
Exon2	17488 – 18169	682
Group I intron L2449	18170 – 18859	690
Exon3	18860 – 19496	637
