# Supplementary material for: Telomere-to-telomere characterization of rDNA chromosome in the myxomycete Didymium iridis
Source: BMC Mol Cell Biol. 2026 Apr 6;27:30. doi: 10.1186/s12860-026-00587-7 (PMC13182076; doi:10.1186/s12860-026-00587-7)
Supplement: Supplementary file 5 — Supplementary Material 5 [file 12860_2026_587_MOESM5_ESM.docx]

**Supplementary information**

The Supplementary Material for this article can be found online at:

**SUPPLEMENTARY FIGURE S3**.

RNA mapping onto *D. iridis* rDNA. (**A**) Combined Illumina library sequencing reads (amoeba, microcyst, flagellate, and plasmodium) are mapped onto the complete rDNA. Note that coverages are displayed as log_10_ values. (**B**) RNA read mappings at separate life-stages; amoeba, microcyst, flagellate, plasmodium. See legend to Figure 4 for abbreviations. Note that coverages are displayed as log_10_ values.
